# Extracellular Competing Endogenous RNA Networks Reveal Key Regulators of Early Amyloid Pathology Propagation in Alzheimer’s Disease

**DOI:** 10.3390/ijms26083544

**Published:** 2025-04-09

**Authors:** Misael Leonardo López-Cepeda, Andrea Angarita-Rodríguez, Alexis Felipe Rojas-Cruz, Julián Pérez Mejia, Robin Khatri, Michael Brehler, Eduardo Martínez-Martínez, Andrés Pinzón, Andrés Felipe Aristizabal-Pachon, Janneth González

**Affiliations:** 1 Departamento de Nutrición y Bioquímica, Facultad de Ciencias, Pontificia Universidad Javeriana, Bogotá 110231, Colombia; misael.lopez@javeriana.edu.co (M.L.L.-C.); maria.angaritar@javeriana.edu.co (A.A.-R.); alexis_rojasc@javeriana.edu.co (A.F.R.-C.); jandres.perezm@javeriana.edu.co (J.P.M.);; 2 Laboratorio de Bioinformática y Biología de Sistemas, Universidad Nacional de Colombia, Bogotá 111321, Colombia; 3 Institute of Medical Systems Bioinformatics, University Medical Center Hamburg-Eppendorf, 20251 Hamburg, Germany; 4 Laboratory of Cell Communication & Extracellular Vesicles, Instituto Nacional de Medicina Genómica, Mexico City 14610, Mexico

**Keywords:** Aβ pathology, amyloidogenic pathway, competitive endogenous RNA network (ceNET), exosome, miRNA

## Abstract

Extracellular vesicles (EVs) are small capsular bodies released by cells, mediating responses in intercellular communication. The role of EVs in Aβ pathology spreading in the Alzheimer’s disease (AD) brain has been evidenced, although whether this occurs due to the co-transportation of Aβ peptides or contribution of other factors, such as EV-associated transcripts, remains uncertain. In vitro studies of miRNA cargo in neuron-derived extracellular vesicles (NDEVs) show that Aβ hyperexpression alters the transcriptomic profile; however, it is not clear to what extent this causes changes at the organ level. By utilizing datasets from published studies, we generated competing endogenous RNA (ceRNA) networks for miRNAs co-expressed in NDEVs and the brain in different stages of pathology, using both an APP overexpressing neuronal model (in vitro) and brain cortices from 6- and 9-month-old APP/PSEN1 mice (in vivo). Networks integrating information from mRNAs, lncRNAs, and circRNAs showed two candidate lncRNAs (Kcnq1ot1 and Gm42969) and a circRNA (Pum1), while enrichment analyses detected that NDEVs miRNAs signal to other CNS cells and that this signal can be disrupted by Aβ pathology, contributing to the loss of long-term potentiation seen in early AD.

## 1. Introduction

Increased deposition of Aβ peptides in the brain is an early indicator of Alzheimer’s disease (AD) [1] and is associated with synaptic disruption, as well as a decline in neuronal viability and function [2]. The amyloid hypothesis suggests that this imbalance is the main contributing factor to AD pathogenesis, triggering other pathophysiological events, such as p-tau accumulation [3], neuronal dysfunction, and death. The limited success of therapies targeting Aβ load has led to criticism of this hypothesis; however, no viable alternative currently exists. Aβ pathology is associated with the spread of AD, synaptic loss, neuronal viability decline, and neuroinflammation, but the full spectrum of dysfunction and what extent it contributes to disease progression remains unknown. Furthermore, the observed spread of Aβ pathology in the brain cannot be fully explained by Aβ infective properties [4], suggesting that the contribution of other factors should be investigated.

Apart from synapses, other forms of intercellular communication play essential roles in the brain (non-synaptic transmission) [5] and modulation of neuroinflammation functions mediated by cytokines have been described previously [6,7]. Disruption of such communication mechanisms could be in addition to cellular stress, a secondary event contributing to pathological spreading. For instance, astrocytes under lipotoxic stress increase ceramide production releasing higher amounts of cytokines that in turn increase tau and Aβ pathology in exposed neurons [8,9,10]. Although neurons are not typically considered major contributors to neuroinflammation, Aβ pathology can potentially disrupt other non-synaptic intercellular communication originating from the affected neurons. This is particularly the case for extracellular vesicle (EV)-mediated communication, which has been proposed to play a role in cellular waste elimination, immunomodulation, neurogenesis, memory encoding and maintenance, and the formation of neuronal circuits [11,12,13]. This is supported by the intracellular accumulation sites of Aβ peptides (early endosomes), which coincide with extracellular vesicle sites of origin, particularly exosomes [14]. Consequently, the extracellular vesicle-mediated toxic effects of Aβ pathology could represent an additional layer of dysregulation independently contributing to Aβ spreading, tau pathology, and neural circuit dysfunction in AD.

This disruption may explain the altered EV profiles observed both in AD brain [15] and in the conditioned media of neurons exposed to Aβ pathology [16]. In fact, EVs have been proposed as facilitators of AD pathology spreading in AD, with their secretion suppression via Neutral sphingomyelinase 2 (nSMase2) blockade as a therapeutic approach to halt pathology spreading in mouse models [17]. Such treatments could interfere with EVs’ normal physiological roles, potentially disrupting EVs from microglia and neurons that are not affected by Aβ pathology. Notably, these EVs have been found to promote Aβ clearance via co-secretion of the Insulin-Degrading Enzyme (IDE) within EVs [18,19]. Furthermore, although several studies have reported upregulation of different amyloid pathway components in the proteome and metabolome of EVs in AD, these findings insufficiently explain the spreading of Aβ pathology observed.

Recent findings in the transcriptome of neuron-derived extracellular vesicles (NDEVs), secreted under Aβ pathology conditions, suggest a role for vesicle-associated miRNA in the spreading of Aβ pathology. One study demonstrated that EVs derived from Neuro2a (N2A) cells, a widely used mouse neuroblastoma cell line serving as a neuronal model that expressed Swedish APP mutation (swAPP) to simulate Aβ pathology [16], exhibited a miRNA expression profile notably lacking mmu-miR-185-5p. This led to the deactivation of a sponging mechanism targeting the APP gene in recipient neurons, inducing an APP upregulation via a differentially expressed miRNA [16]. Such changes at the distance are supported in vivo with evidence of sequential dysregulation of transcriptomic profiles [20,21,22], suggesting that miRNA differential expression in cell-specific EVs could contribute to those observations. However, a more systematic definition of NDEV miRNA contribution in pathology propagation is lacking in an in vivo context.

Competing endogenous RNA networks (ceNETs) analyze miRNA interactions with target transcripts based on endogenous RNA interference [23], providing a framework to infer transcriptome regulation and, consequently, cellular function at a systemic level. According to this hypothesis, miRNA expression can be described in terms of competing endogenous RNAs (ceRNAs), which include any coding or non-coding RNA that physically interacts with a miRNA. In this model, if miRNA upregulation occurs, it indicates that sponging mechanisms are off, whereas downregulation suggests active ceRNA sponging. Thus, the model is useful for understanding cell function in different conditions by examining mRNA expression associated with a ceNET. Complex ceNETs based on intracellular expression have been constructed, leading to the proposal of new pharmacological targets [24,25,26]. We propose that miRNAs play a central role in modeling ceNETs of vesicle origin because their small size theoretically makes them more likely to be packaged into vesicles, the higher number of RNA-protein interactions described for miRNA-specific vesicle packaging compared to other RNAs [27], and the critical dependency of RNA interference mechanisms in miRNA expression. Thus, we consider the ceRNA hypothesis an adequate approach for predicting the impact of Aβ pathology on intercellular communication mechanisms mediated by NDEVs at the RNA level.

**Figure 1:**
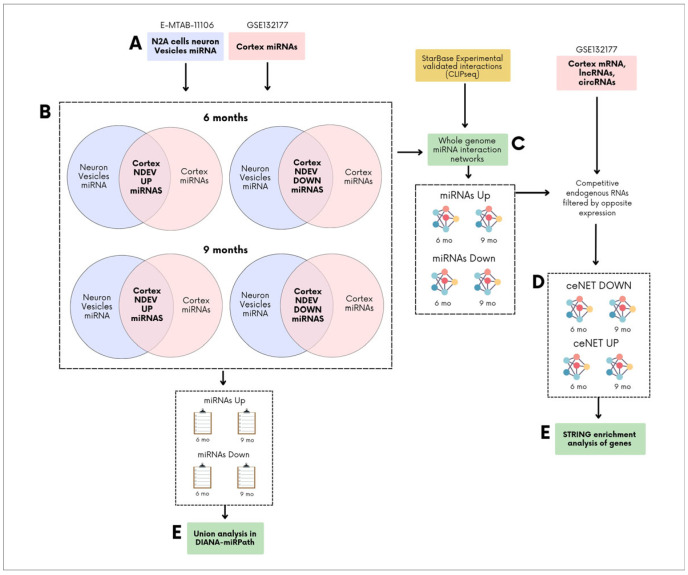
General overview of competing endogenous RNA networks (ceNETs) construction. (**A**) Upregulated and downregulated miRNAs from neuron-derived extracellular vesicles (NDEVs), of N2A cells under APP hyperregulation (E-MTAB-11106) (blue) [16], and from brain cortices of 6- and 9-month-old APP/PSEN1 mice (GSE132177) (pink), were first selected as a subset [28,29]. (**B**) Upregulated miRNAs from brain cortices were compared with those from neuronal vesicles to identify shared miRNAs that are upregulated in both neuronal vesicles (from neurons under APP hyperregulation) and the cortex. The same analysis was performed for downregulated miRNAs. (**C**) The overlapping upregulated or downregulated miRNAs were used as an input to build whole-genome ceNETs for each set of overlapping miRNAs, using crosslinking and immunoprecipitation sequencing (CLIP-seq) miRNA-target interactions taken from ENCORI [31]. (**D**) Upregulated ceNETs were constructed by filtering mRNA, long non-coding RNA (lncRNA), and circular RNA (circRNA) interactions based on cortex expression data (GSE132177) (pink) [28,29], selecting only those with opposite expression patterns. The same approach was applied to generate downregulated ceNETs. (**E**) miRNAs and ceNET mRNA were then analyzed using DIANA-miRPath v4.0 and STRING v12.0 [32,33], respectively.

Here, we constructed comprehensive ceNETs representing the brain’s response to differentially expressed miRNAs from NDEVs isolated from neurons under amyloidogenic stress, to model the contribution of the EV transcriptome in amyloid and Alzheimer’s disease (AD) pathology spreading. Considering neuronal signaling dysregulation, we selected vesicle-detected miRNAs produced by neurons under amyloid hyperregulation in vitro [16]. After filtering this expression based on in vivo datasets from previous studies of APP/PSEN1 mouse brains [28,29], we constructed genome-wide ceRNA networks supported by crosslinking and immunoprecipitation sequencing (CLIP-seq) interactions, which were later filtered based on brain ceRNA expression data [28,29] (Figure 1). We leveraged miRNA, mRNA, lncRNA, and circRNA expression data available in the same APP/PSEN1 mouse model at different stages (6 and 9 months), sourced from earlier studies, to systematically examine the simultaneous contribution of different ceRNAs to the spread of amyloid and Alzheimer’s pathology at early-stage (6 months) and later-stage (9 months). lncRNAs previously associated with AD, such as NEAT1 [30], were found to be associated with later-stage of dysregulation and two candidate lncRNAs (Kcnq1ot1 and Gm42969) and a circRNA (Pum1) were identified. Taken together, our systematic approach provides the first comprehensive mapping of NDEV-associated miRNA networks in early and established amyloid pathology, identifying novel regulatory elements that may represent key drivers of disease progression and potential therapeutic targets.

## 2. Results

**Figure 2:**
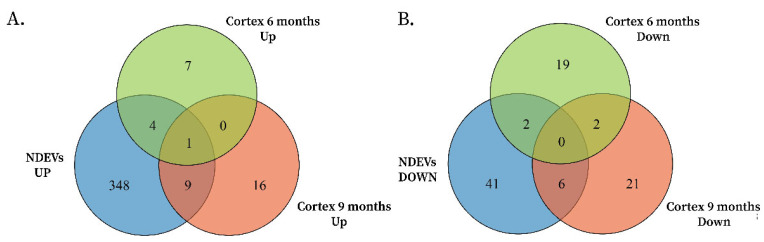
(**A**) Overlap analysis identified 5 upregulated miRNAs shared between 6-month-old APP/PSEN1 cortex and neuronal vesicles under amyloid stress and 10 upregulated miRNAs shared between 9-month-old APP/PSEN1 cortex and neuronal vesicles. mmu-miR-369-5p was the miRNA found upregulated in all groups. (**B**) A similar analysis for downregulated miRNAs found 2 miRNAs in the 6-month-old cortex-vesicle overlap and 6 miRNAs in the 9-month-old cortex-vesicle overlap.

ceNETs were constructed to model how miRNAs released within EVs from neurons under amyloid stress (Aβ peptide hyperregulation) modulate the brain expression profile. Networks were built using differentially expressed miRNAs from APP/PSEN1 mouse brain cortex, filtered based on their equal dysregulation in EV produced by stressed neurons (e.g., miRNAs upregulated in both the cortex and neuronal vesicles were retained) (Figure 1). We leveraged the availability of 6- and 9-month-old cortex miRNA expression profiles to model age-dependent processes. This approach assumes that amyloid pathology stressed neurons maintain a stable release of dysregulated miRNAs within EVs, allowing us to filter cortical miRNAs of different ages using the same sets of upregulated or downregulated neuronal vesicle miRNAs. As a result, we obtained distinct miRNA sets upregulated or downregulated in both neuronal vesicles and APP/PSEN1 cortex at each age (Figure 1 and Figure 2, Table 1). These miRNA sets were used as input for constructing a whole-genome interaction network using CLIP-seq interactions reported in ENCORI [31] of the miRNAs sets with competing endogenous RNAs (mRNAs, lncRNAs and circRNAs). Exploratory enrichment analysis of miRNA sets with miRPath v4.0 [33] evidenced neuron projections dysregulation and other disruptions, which were later explored with STRING v12.0 enrichment analysis [32].

**Figure 3:**
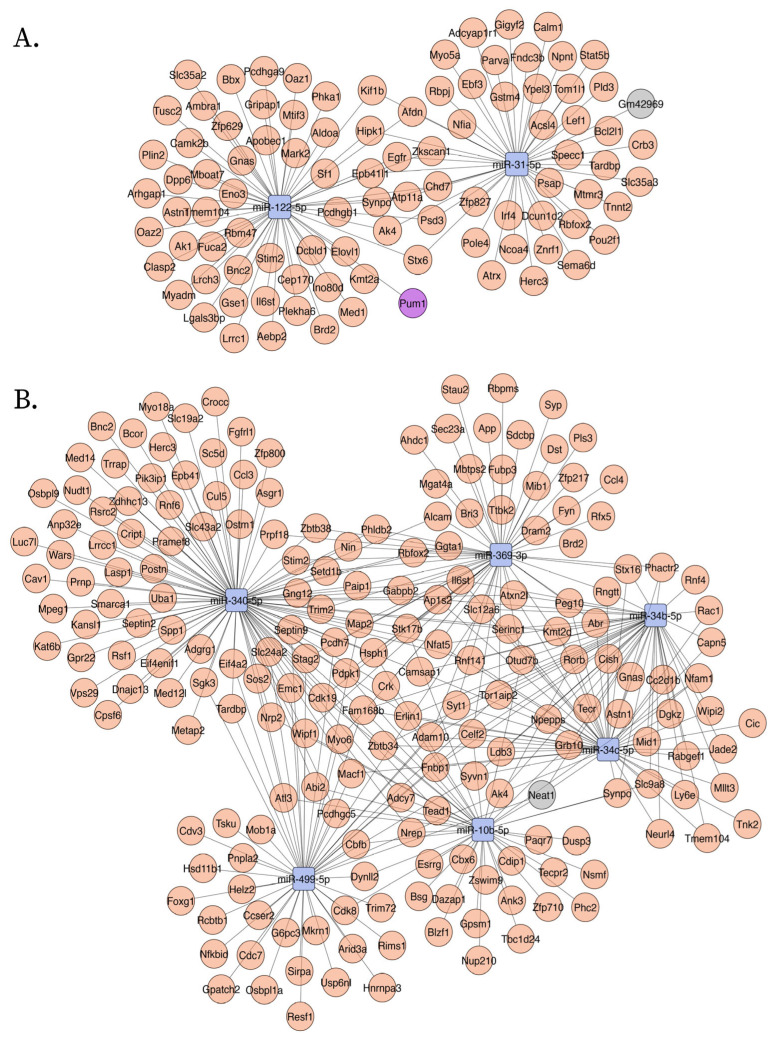
Upregulated ceNETs show upregulated genes and associated regulatory transcripts in the APP/PSEN1 mouse brain at 6 months (**A**) and 9 (**B**) months of age. Networks were generated using Cytoscape (version 3.10.3) with a prefuse force-directed layout. Nodes were manually adjusted for clarity. Downregulated miRNAs: blue; upregulated mRNA: red; lncRNAs: gray; circRNA: purple.

**Figure 4:**
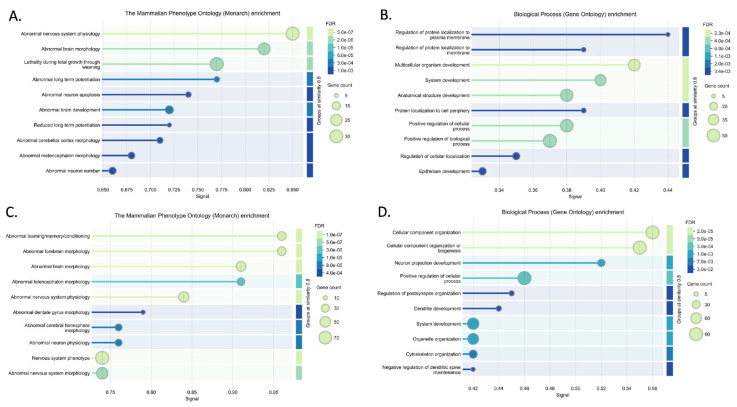
Enrichment analysis results for upregulated networks (UP ceNETs) in Mammalian Phenotype Ontology (Monarch) and GO biological processes, performed using the STRING v12.0 database [32]. Significant Monarch (**A**) and GO biological process (**B**) terms for the 6-month-old UP ceNET, and Monarch (**C**) and GO biological process (**D**) terms for the 9-month-old UP ceNET.

Mechanisms of disease activated in AD were explored using upregulated ceNETs (UP ceNETs) (Figure 3), built by filtering whole genome ceRNA interactions of 6- and 9-month-old downregulated cortex miRNAs, with 6- and 9-month-old upregulated cortex ceRNA expression information. ENCORI reported interactions for every downregulated miRNA in miRNA sets (Tables S1 and S2). Enrichment analysis of Mammalian Phenotype Ontology (Monarch) terms revealed abnormalities in nervous system physiology, brain morphology, long-term potentiation (LTP), and neuron apoptosis at early-stage and abnormalities forebrain and telencephalon morphology, nervous system physiology and learning/memory/conditioning at later-stage. Abnormalities in dentate gyrus morphology and neuron physiology were also found in later-stage, suggesting continuity of early LTP abnormalities in a key region for memory formation (Figure 4). In addition, GO biological process enrichment analysis showed regulation of protein localization to plasma membrane, multicellular organism development and system development, as upregulated processes in early-stage, and cellular component organization and neuron projection development as upregulated in later-stage. Interestingly, negative regulation of dendritic spine maintenance was also activated but with a weaker signal in later-stage (Figure 4). Additional enrichment analysis of subcellular localization (compartments) revealed ruffle, intracellular organelle, organelle, and recycling endosome in early-stage and actin cytoskeleton, intracellular organelle, and neuron to neuron synapse in later-stage. GO cellular component enrichment showed ruffle, contractile fiber, and endosome terms in early-stage and actin cytoskeleton, neuron to neuron synapse, and postsynaptic density terms in later-stage (Figure S1).

**Figure 5:**
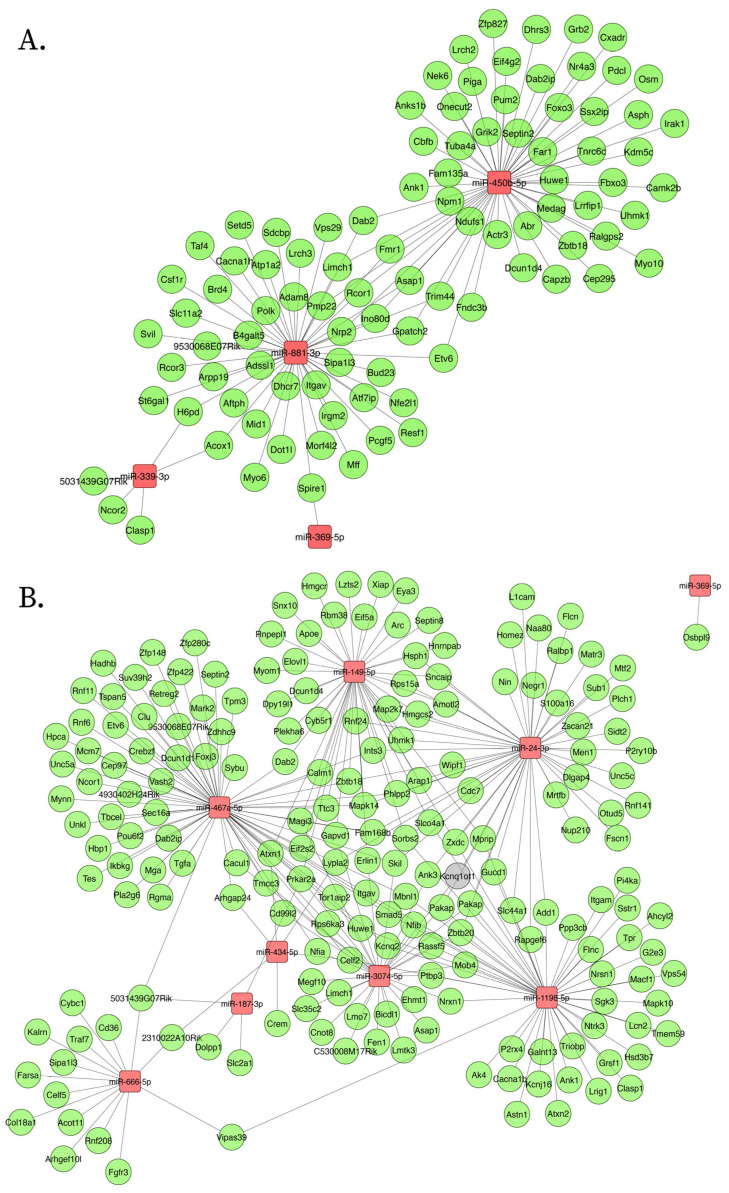
Downregulated ceNETs show downregulated genes and associated regulatory transcripts in the APP/PSEN1 mouse brain at 6 months (**A**) and 9 (**B**) months of age. Networks were generated using Cytoscape (version 3.10.3) with a prefuse force-directed layout. Nodes were manually adjusted for clarity. Upregulated miRNAs: red; downregulated mRNA: green; lncRNA: gray.

**Figure 6:**
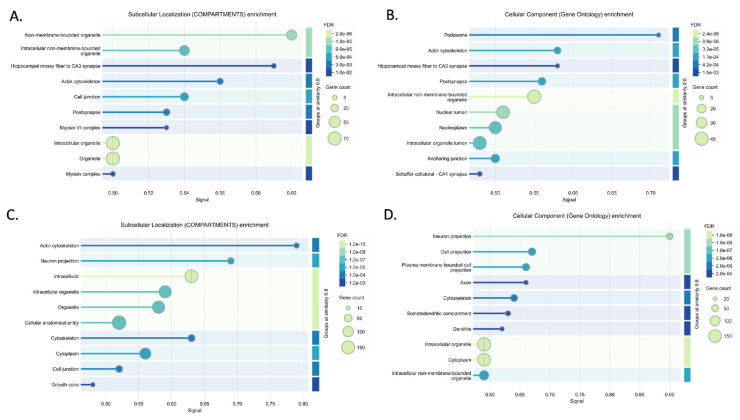
Enrichment analysis results for downregulated networks (DOWN ceNETs) in subcellular localization (compartments) and GO cellular components, performed using the STRING v12.0 database [32]. Significant compartments (**A**) and GO cellular component (**B**) terms for the 6-month-old DOWN ceNET and compartments (**C**) and GO cellular components (**D**) terms for the 9-month-old DOWN ceNET.

Conversely, homeostasis mechanisms maintained by healthy neurons could be interrupted in the AD brain by abnormal activation of NDEVs miRNAs. Such mechanisms were explored using DOWN ceNETs, constructed by filtering whole genome ceRNA interactions of 6- and 9-month-old upregulated cortex miRNAs, with downregulated cortex 6- and 9-month-old ceRNA expression information, thus obtaining DOWN ceNETs (Figure 5). ENCORI reported interactions for all upregulated miRNA but mmu-miR-1983 in 6-month set and mmu-miR-99b-3p in 9-month set (Tables S3 and S4). Enrichment subcellular localization (compartments) terms of such dysregulation showed early-stage disruption in genes of non-membrane-bounded organelles, hippocampal mossy fiber to CA3 synapse, actin cytoskeleton and cell junction, and later-stage disruption of actin cytoskeleton, neuron projections, intracellular organelle, cell junction, and cytoskeleton (Figure 6). GO cellular component enrichment showed disruptions in the podosome, actin cytoskeleton, hippocampal mossy fiber to CA3 synapse, postsynapse and non-membrane bound organelle at early-stage, and neuron projection, cell projection, plasma membrane bound cell projection and axon at later-stage (Figure 6). Interestingly, GO cellular component enrichment evidenced dysfunction in the somatodendritic compartment, dendrite, and non-membrane bounded cell organelle at the later-stage, and subcellular localization (compartments) terms showed enrichment for myosin complex in early-stage (Figure 6).

Further enrichment results for Monarch terms in DOWN ceNETs showed abnormal cell differentiation, prenatal growth retardation, and lethality during fetal growth in the early-stage, and abnormal exploration in a new environment, abnormal response to novelty, and abnormal learning/memory/conditioning in the later-stage. GO biological process enrichment showed regulation of organelle organization, regulation of cellular component organization, and positive regulation of cellular component organization in the early-stage, while for the later-stage enrichment showed positive regulation of cellular component organization, neuron projection development, and regulation of cellular localization. Interestingly, GO biological process enrichment identified actin cytoskeleton organization enrichment in the early-stage and homeostasis of number of cells and positive regulation of protein modification process in the later-stage (Figure S2). Later-stage DOWN ceNET at 9 months was the only network reporting two miRNAs in other studies: mmu-miR-149-5p and mmu-miR-24-3p, which hyperregulated in APP/PSEN1 mouse hippocampus (GSE138382).

These results suggest that NDEVs networks operate in key regions and processes implicated in AD, particularly at the CA3 synapse and during LTP. This signal predominantly targets brain cells, particularly neurons, which align with the enrichment results of TISSUE terms indicating significant enrichment in the brain and nervous system areas across all networks in both early- and later-stage (Figure S3). STRING enrichment analysis of subcellular localization (compartments), cellular component (GO), and Monarch terms were also performed with upregulated and downregulated, unselected genes for both stages (early and late) and results were compared with corresponding ceNETs (Figure S4). Subcellular localization (compartments) results showed dominance of intracellular locations in unselected downregulated genes, not seen in early-stage DOWN ceNET, while enrichment of hippocampal mossy fiber to CA3 synapse, actin cytoskeleton, cell junction, postsynapse and myosin terms was exclusive of 6-months DOWN ceNET (Figure S5). A similar pattern was found in later-stage subcellular localization (compartments) enrichment which showed exclusivity in actin cytoskeleton and growth cone terms for 9-months DOWN ceNET (Figure S6). For upregulated genes, recycling endosome term was found exclusively in early-stage UP ceNET and not in unselected mRNAs (Figure S7). Notably, GO cellular component terms of unselected downregulated genes showed no evidence of podosome, actin cytoskeleton, hippocampal mossy fiber to CA3 synapse, postsynapse, and schaffer collateral–CA1 synapse enrichments seen in their selected early-stage DOWN ceNET counterpart (Figure S8). Furthermore, the later-stage GO cellular component enrichment revealed axon and dendrite term exclusivity in late stage selected DOWN ceNET (Figure S9). When GO cellular component terms not notable differences were found in upregulated genes in early-stage, however negative regulation of dendritic spine maintenance was not found in the unselected genes in later-stage (Figures S10 and S11), which is consistent with previous results. Monarch enrichment analysis again reported no profound differences between the upregulated unselected genes and the early-stage UP ceNET (Figure S12), but in the later-stage exclusiveness for nervous system physiology, and abnormal forebrain, telencephalon and, interestingly, dentate gyrus morphology were detected in later-stage UP ceNET (Figure S13). Collectively, these results support a remarkably coherent miRNA signal exclusive for genes selected in NDEV ceNET modifying LTP and hippocampus synapses in early-stage and neuron projections in later-stage.

## 3. Discussion

This study demonstrates that EVs derived from amyloid-stressed neurons act as molecular messengers, mediating intercellular communication changes that influence both early and later stages of amyloid pathology, integrating in vitro data from amyloid-overexpressing N2A cells with in vivo data from APP/PSEN1 mouse brain cortices at different stages (6 and 9 months). From this, we retrieved comprehensive expression profiles of miRNAs, mRNAs, and lncRNAs to construct ceRNA networks. These networks provide insights into unraveling systemic transcriptomic disturbances triggered by EV-mediated RNA transfer under amyloid stress.

Neuronal EVs have increasingly been recognized as crucial players in Aβ pathology propagation [17]. However, prior research largely focused on the protein cargo within NDEVs, particularly amyloid-β (Aβ) peptides [14,17,34]. We demonstrate that vesicle RNA content, specifically dysregulated miRNAs, has an equally important role. One of the key findings of our study is the significant dysregulation of neuronal projections seen in later-stage and hippocampal mossy fiber to CA3 synapse in early-stage (Figure 6, Figure S8 and Figure S9). These enrichment results were not observed in either UP ceNETs or unselected downregulated mouse brain samples from 6- and 9-month-old APP/PS1 mice, which is consistent with our findings. Furthermore, Arc, a gene previously proposed as critical for memory consolidation and synaptic plasticity [11], was found in 9-month-old DOWN ceNET negatively regulated by mmu-miR-149-5p. These results support the relevance of LTP and episodic memory in hippocampal neurons in this axis, suggesting NDEVs from neurons affected by Aβ pathology have a contribution to the impairments seen at mossy fiber–CA3 synapses in the early-stage of AD, which is in line with our finding of this dysregulation in 6-month-old mice.

In addition, the abnormalities in LTP and dentate gyrus evidenced as Monarch enriched terms of UP ceNETs is particularly noteworthy, because no evidence of this was found on their downregulated counterparts (Figure 5, Figure 6 and Figure S2, see Monarch), obtaining the same result with independent datasets. Such dysregulation was also not tracked in enrichment analysis of upregulated 6- and 9-month-old unselected mRNAs (Figures S12 and S13), confirming this enrichment is unique in ceNET selected mRNAs. LTP abnormalities were specifically identified in upregulated ceNETs from 6-month-old APP/PSEN1 mice, consistent with LTP being a process known to be dysregulated early in AD [35]. Notably, no previous studies have reported an association between vesicle neuron miRNA dysregulation by Aβ pathology and LTP dysregulation. Taken together, this evidence suggests neuron vesicle miRNA signaling targets LTP in other neurons inside the brain, which is in line with tissue expression enrichments showing high enrichment for brain and central nervous system locations. This is also supported by experimental evidence of neuronal vesicle uptake in vitro in primary neuronal cultures [36]. Such signaling could be dysregulated in early AD indirectly through vesicles from neurons under amyloidogenic stress contributing to early loss of memory.

Beyond miRNAs and coding genes, this work identified lncRNA NEAT1, already implicated in stress granule formation and neuroinflammation, chronic stress responses [25] and a well-known lncRNA in AD [30]. Furthermore, three new regulatory RNAs integrating into these networks were identified further amplifying the complexity of transcriptomic regulation; among these, Kcnq1ot1 was previously associated with promoting proinflammatory markers in lipopolysaccharide (LPS)-induced microglia, and reducing neurological dysfunction in vivo in mice (Xia et al. 2022) [37,38] and Gm42969. Both have not been reported in AD, thus constitute new candidate regulators. Network function suggests that Gm42969 may have a role similar to NEAT1 but during earlier disease stages, potentially making both candidates therapeutic targets of AD intervention. Finally, circRNA Pum1 with a previous role mitigating cerebral ischemia [39] was found in upregulated early-stage ceNET, which may reflect a compensatory response. Taken together, these findings support the ceRNA hypothesis, which posits that non-coding RNAs can compete for miRNA binding, thereby modulating gene expression and contributing to disease progression [23,25].

Together, these results demonstrate how miRNAs from EVs could contribute a role in AD pathology spreading by modulating intercellular communication through dysregulated miRNAs. Our ceRNA network analysis reveals a systemic view of the transcriptomic disturbances triggered by amyloid stress, revealing novel regulatory elements such as lncRNAs (Kcnq1ot1 and Gm42969) and circRNA (Pum1), alongside the well-known lncRNA NEAT1. These networks highlight the dysregulation of key processes, including synaptic function and LTP, which are crucial for memory and are impaired in early AD. The findings also demonstrate stage-specific changes, with the later-stage involving alterations in neuron projection development. Taken together, these insights not only deepen our understanding of the molecular mechanisms driving AD progression but also identify potential therapeutic targets for disrupting Aβ pathology and neuroinflammation.

## 4. Materials and Methods

### 4.1. Data Extraction

Data were obtained from previously published, publicly available sources [16,28,29,40,41]. Expression profiles of miRNA from EVs from neurons under amyloid stress were taken from N2A cell cultures transfected with human APP overexpression plasmid (E-MTAB-11106) [16,40]. N2A cells are a widely used neuronal model in neurodegenerative disease research [14]. Expression profiles of miRNA, mRNA, lncRNAs and circRNAs from 6-month and 9-month-old mouse brain cortex with amyloid pathology, were taken from APP/PSEN1 mice (GSE132177) [28,29,41]. lncRNA and mRNA differential expression was obtained using a new tuxedo protocol [42], with reference transcriptome from Gencode using GSE132177 libraries, while circRNA and miRNA expression was obtained from previously reported expression profiles. APP/PSEN1 is a amyloid pathology mouse model showing one of the highest resemblances with AD [43]. Other datasets used for validation included GSE138382, GSE157239, GSE155700, and GSE46579, which were obtained from Gene Expression Omnibus [41,44,45].

### 4.2. Development of the Competing Endogenous RNA Network

Upregulated and downregulated miRNAs from NDEVs were compared with those from the cortex using a custom Python 3.12 script. miRNAs shared between NDEVs and cortex were used to construct ceNETs: UP miRNAs formed downregulated ceNETs, while DOWN miRNAs formed upregulated ceNETs.

Genome-wide CLIP-seq validated miRNA interactions from ENCORI [31] were first used to build ceRNA networks. These networks were then filtered using circRNA, lncRNA, and mRNA expression data from 6- and 9-month-old mouse cortex, enabling the construction of ceNETs representing early and later pathology.

According to the ceRNA hypothesis miRNA expression depends on ceRNA networks that operate at a cell, or in this case at an organ, level and if a miRNA is upregulated, ceRNA mechanisms regulating its expression are off. Contrarily, if miRNA is downregulated, ceRNA mechanisms are on, where a ceRNA is any transcript with demonstrated interaction with a miRNA. Thus, for network construction, UP miRNA interactions were filtered out with the expression data of downregulated ceRNA only. Contrarily, DOWN miRNAs interactions filtering was carried out with upregulated ceRNA expression data. Results were recreated with Cytoscape 3.10.3 [46].

### 4.3. Enrichment Analysis

DIANA-miRPath v.4 pathway union analysis [33] (Table S5) was employed in each miRNA set to explore the functional annotation of miRNAs found in both mouse brain cortex and NDEVs. For miRPath v.4, experimental (TarBase v 8.0 and miRTarBase 2022), and computational (TargetScan and microT-CDS) databases were explored (Tables S6–S9).

Furthermore, gene enrichment analysis was performed using the STRING v12.0 database following network construction [32]. A 0.05 False Discovery Rate (FDR) threshold was applied for statistical significance and only terms with a minimum signal strength of 0.01 were included. Terms were considered only if at least two genes were present in the network. To ensure clarity, enriched terms were displayed separately, without merging similar terms, and were grouped based on a similarity threshold of ≥0.2.

Finally, STRING enrichment analysis was conducted on upregulated and downregulated mRNAs from both 6- and 9-month-old APP/PSEN1 mouse brains using the same parameters. This allowed for direct comparison with the network-based STRING enrichment results.

## Figures and Tables

**Table 1:** List of mouse brain miRNAs selected for ceNET construction. miRNA selection was based on consistent dysregulation of the miRNA between the APP/PSEN1 mouse cortex (at 6 or 9 months of age) and an Aβ-stressed neuronal model with APP hyperregulation. The table includes normalized expression levels (expressed as the median of ratios from DESeq2) for each miRNA and the top five enriched GO terms (all) for each set. Pathway union enrichment was performed using miRPath v4.0, with computational predictions from TargetScan and microT-CDS databases. APP: APP/PS1 mouse, WT: wild type mouse.

Symbol	Expression	WT	APP	Functional Annotation
mmu-miR-339-3p	**miRNA**: Up **ceNET**: Down 6 months	86	110	Protein binding, mitochondrion, endoplasmic reticulum membrane, protein kinase binding and protein homodimerization activity
mmu-miR-369-5p		2999	3361	
mmu-miR-450b-5p		10	18	
mmu-miR-881-3p		17	33	
mmu-miR-1983		335	423	
mmu-miR-31-5p	**miRNA**: Down **ceNET**: Up 6 months	195	160	Nucleus, cytoplasm, protein binding, neuron projection and metal ion binding
mmu-miR-122-5p		42	24	
mmu-miR-24-3p	**miRNA**: Up **ceNET**: Down 9 months	21,261	23,968	Protein binding, flavonoid glucuronidation, xenobiotic glucuronidation, protein polyubiquitination and actin cytoskeleton organization
mmu-miR-99b-3p		785	905	
mmu-miR-149-5p		2240	2806	
mmu-miR-187-3p		1348	1655	
mmu-miR-369-5p		2962	3232	
mmu-miR-434-5p		104,812	116,640	
mmu-miR-467a-5p		2758	3252	
mmu-miR-666-5p		1165	1441	
mmu-miR-1198-5p		1258	1428	
mmu-miR-3074-5p		21,228	23,940	
mmu-miR-10b-5p	**miRNA**: Down **ceNET**: Up 9 months	218	161	Nucleus, protein binding, cytoplasm, nucleoplasm, and positive regulation of transcription by RNA polymerase II
mmu-miR-34b-5p		30	12	
mmu-miR-34c-5p		7020	4872	
mmu-miR-340-5p		7643	6290	
mmu-miR-369-3p		1674	1456	
mmu-miR-499-5p		187	154	

## Data Availability

These data were derived from the following resources available in the public domain: (1) ArrayExpress: https://www.ebi.ac.uk/biostudies/ArrayExpress/studies/E-MTAB-11106?query=E-MTAB-11106 (accessed on 5 April 2025); (2) Gene Expression Omnibus: https://www.ncbi.nlm.nih.gov/geo/query/acc.cgi?acc=GSE132177, https://www.ncbi.nlm.nih.gov/geo/query/acc.cgi?acc=GSE138382, https://www.ncbi.nlm.nih.gov/geo/query/acc.cgi?acc=GSE157239, https://www.ncbi.nlm.nih.gov/geo/query/acc.cgi?acc=GSE155700, and https://www.ncbi.nlm.nih.gov/geo/query/acc.cgi?acc=GSE46579 (accessed on 5 April 2025).

## Supplementary Materials

The following supporting information can be downloaded at: https://www.mdpi.com/article/10.3390/ijms26083544/s1.

## Abbreviations

The following abbreviations are used in this manuscript:

ceNET	Competing endogenous RNA network
circRNA	Circular RNAs
lncRNAs	Long non-coding RNAs
LTP	Long-term potentiation
MDPI	Multidisciplinary Digital Publishing Institute
miRNAs	Micro RNAs
mRNA	Messenger RNAs
DOAJ	Directory of open access journals
