# Supplementary material for: Extracellular Competing Endogenous RNA Networks Reveal Key Regulators of Early Amyloid Pathology Propagation in Alzheimer’s Disease
Source: Int J Mol Sci. 2025 Apr 9;26(8):3544. doi: 10.3390/ijms26083544 (PMC12027385; doi:10.3390/ijms26083544)
Supplement: Supplementary file 1 [file ijms-26-03544-s001.zip › Supplementary Figures S1-S13.pdf]

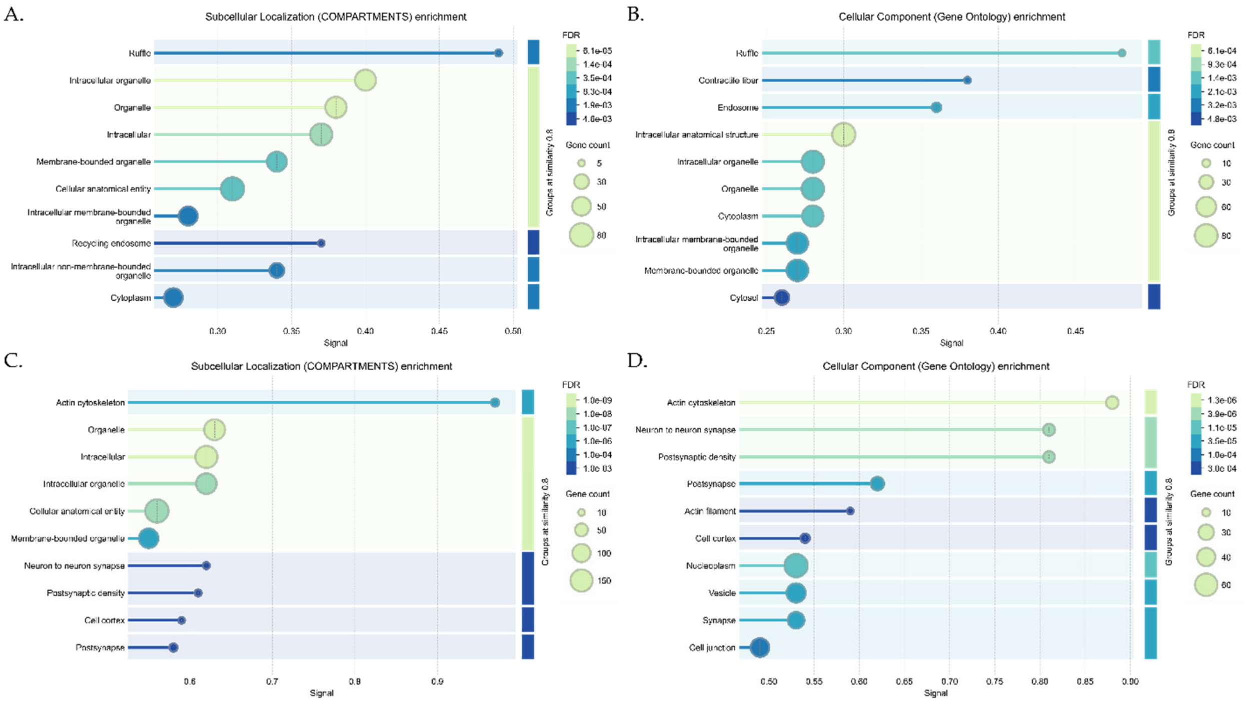

Figure S1. Enrichment analysis results for UP ceNETs in subcellular localization (Compartments) and GO cellular components. Significant compartments (**A**) and GO cellular component (**B**) terms for the 6-month-old UP ceNET, and compartments (**C**) and GO cellular components (**D**) terms for the 9-month-old UP ceNET.

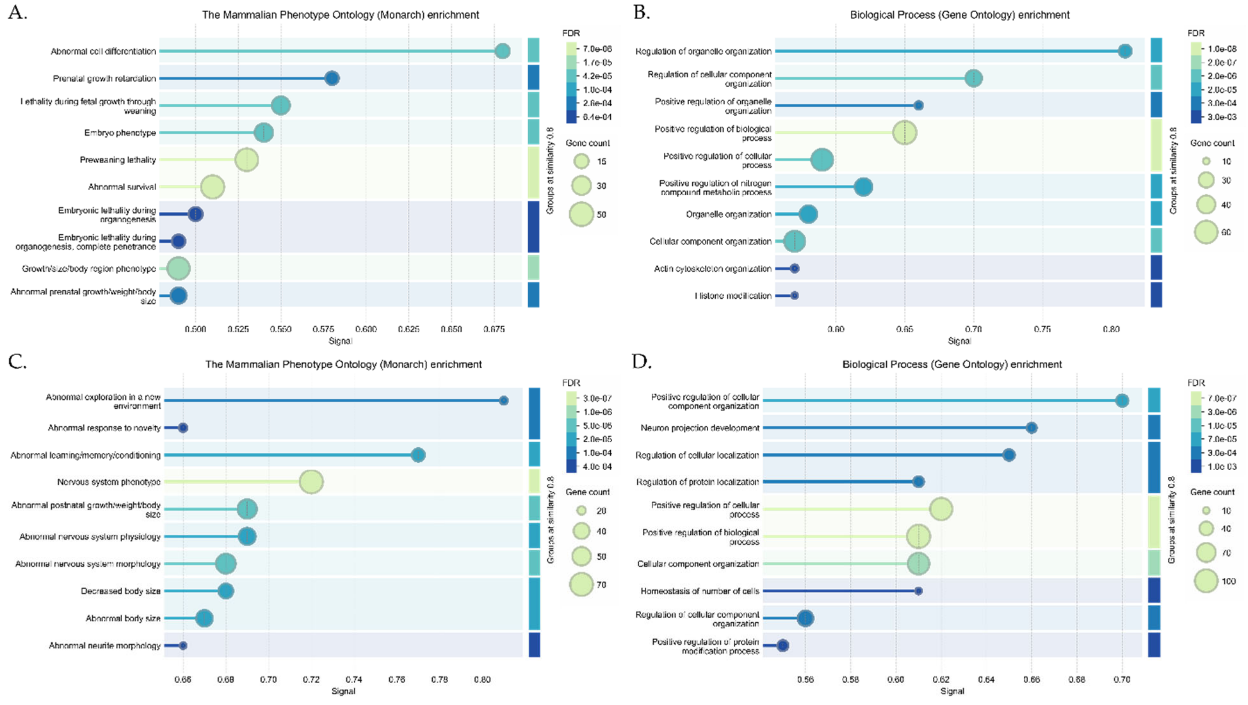

Figure S2. Enrichment analysis results for DOWN ceNETs in Mammalian Phenotype Ontology (Monarch) and GO biological processes. Significant Monarch (**A**) and GO biological process (**B**) terms for the 6-month-old DOWN ceNET, and Monarch (**C**) and GO biological process (**D**) terms for the 9-month-old DOWN ceNET.

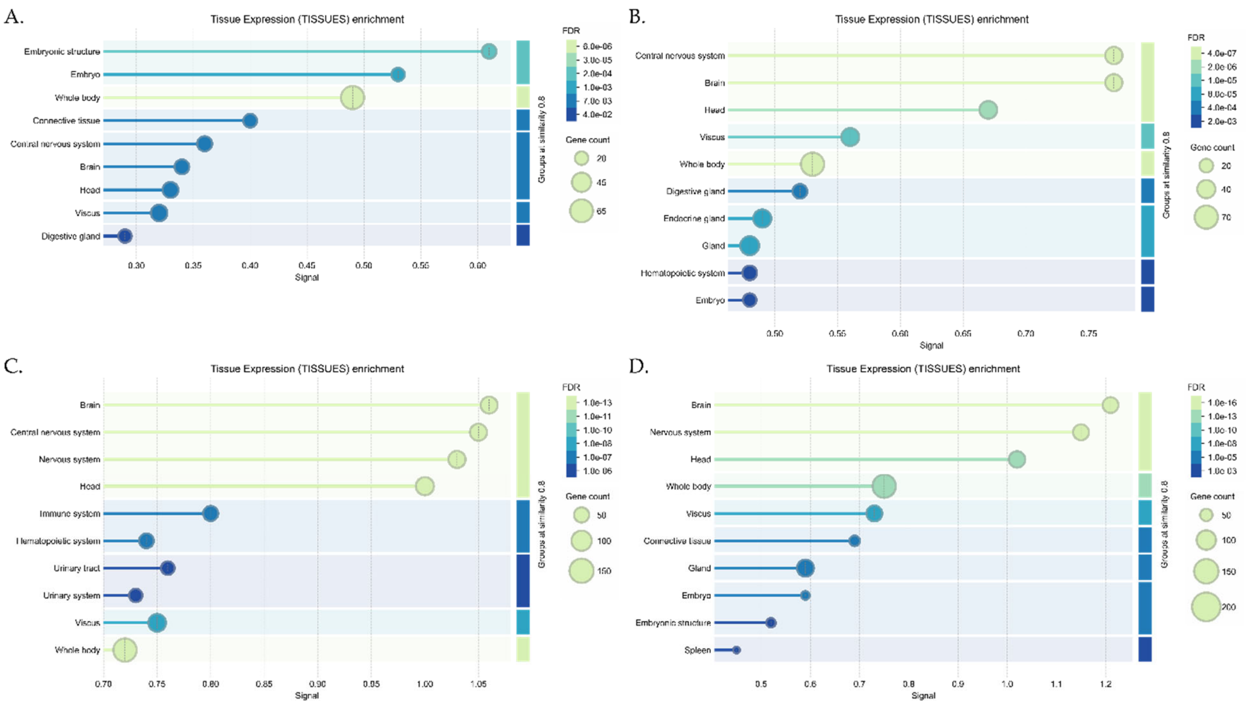

Figure S3. Main enriched tissue expression terms (TISSUES) of UP (**A**) and DOWN (**B**) 6-month ceNETs and UP (**C**) and DOWN (**D**) 9-month ceNETs.

Analysis

Compare enrichment analysis of unselected mRNA vs the appropriateceNET:

List of comparisons

- 6-month DEG Downregulated mRNAs vs 6mo DOWNceNET.
- 9-month DEG Downregulated mRNAs vs 9-mo DOWN ceNET.
- 6-month DEG Upregulated mRNAs vs 6mo UPceNET
- 9-month DEG Upregulated mRNAs vs 9mo UPceNET

Enrichment analysis: 1) Subcellular Localization (COMPARTMENTS), 2) Cellular component (GO), 3) The Mammalian Phenotype Ontology (Monarch)

Figure S4. Comparisons done for enrichment analysis of unselected mRNAs vs ceNET mRNAs.

6-month Downregulated mRNAs vs **6mo DOWN ceNET**

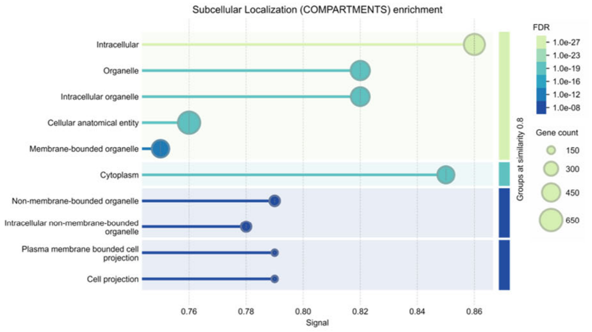

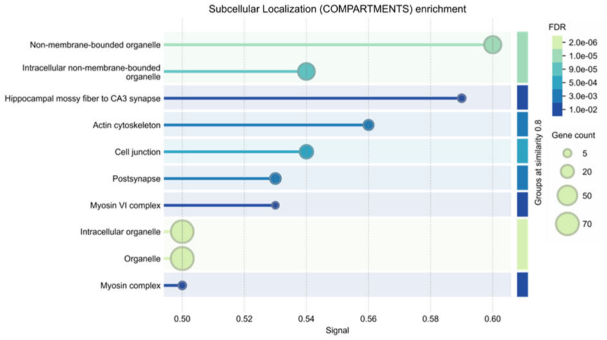

mRNA: unselected 6 mo downregulated

ceNET: 6mo DOWN

Figure S5. Subcellular localization (Compartments) enrichment results: 6-month Downregulated mRNAs vs 6mo DOWN ceNET.

9-month Downregulated mRNAs vs **9mo DOWN ceNET**

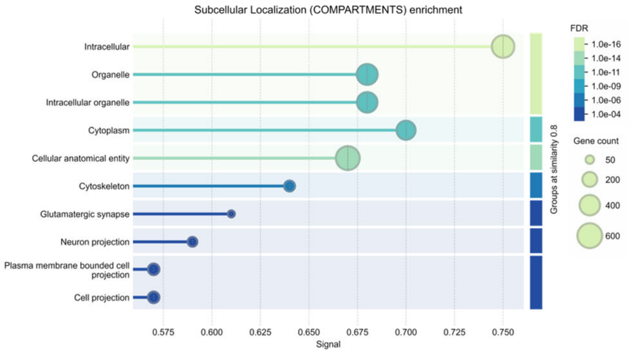

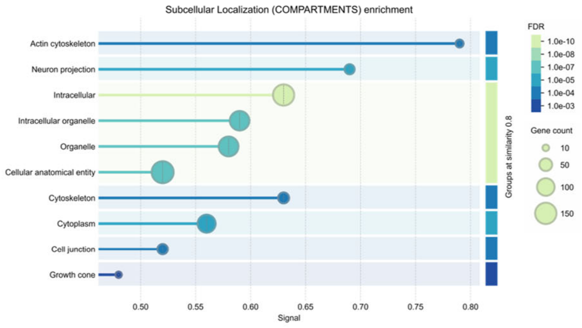

mRNA: unselected 9 mo downregulated

ceNET: 9mo DOWN

Figure S6. Subcellular localization (Compartments) enrichment results: 9-month Downregulated mRNAs vs 9mo DOWN ceNET.

6-month upregulated mRNAs vs **6mo UP ceNET**

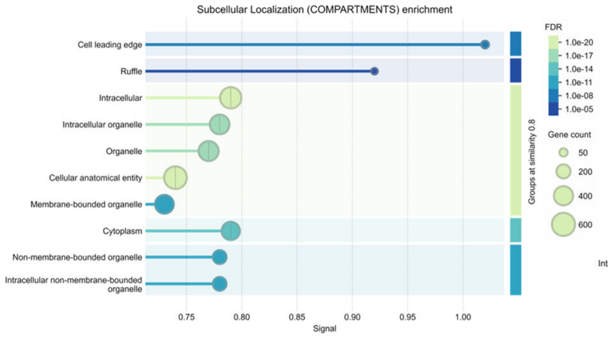

mRNA: unselected 6 mo upregulated

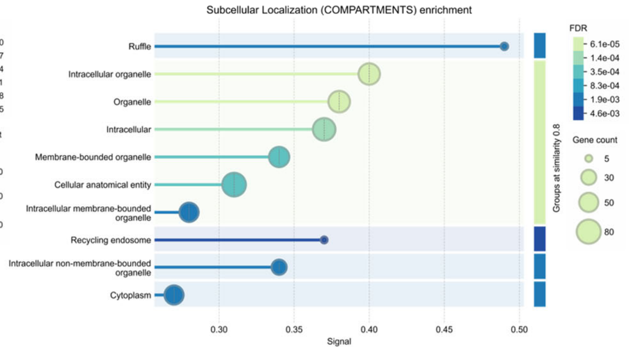

ceNET: 6mo UP

Figure S7. Subcellular localization (Compartments) enrichment results: 6-month upregulated mRNAs vs 6mo UP ceNET.

6-month Downregulated mRNAs vs **6mo DOWN ceNET**

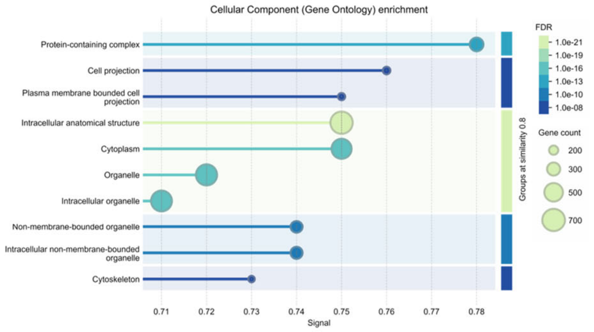

mRNA: unselected 6 mo downregulated

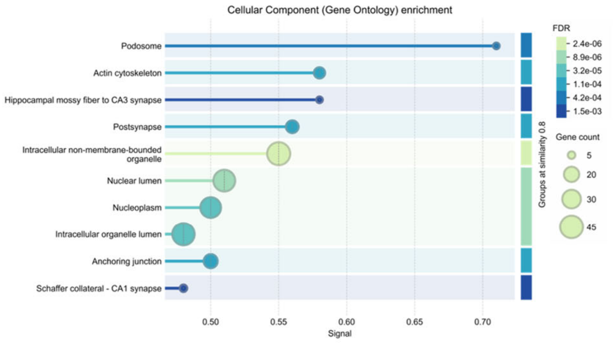

ceNET: 6mo DOWN

Figure S8. Cellular component (GO) enrichment results: 6-month Downregulated mRNAs vs 6mo DOWN ceNET.

9-month Downregulated mRNAs vs **9mo DOWN ceNET**

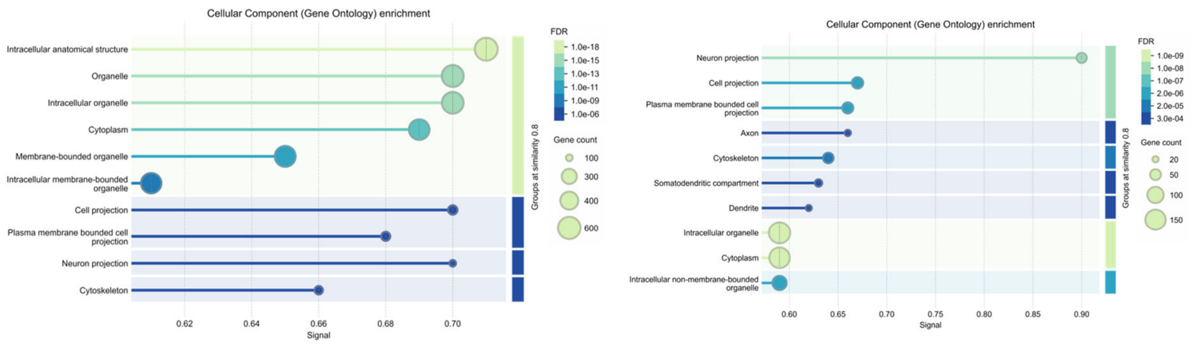

mRNA: unselected 9 mo downregulated

ceNET: 9mo DOWN

Figure S9. Cellular component (GO) enrichment results: 9-month Downregulated mRNAs vs 9mo DOWN ceNET.

6-month upregulated mRNAs vs **6mo UP ceNET**

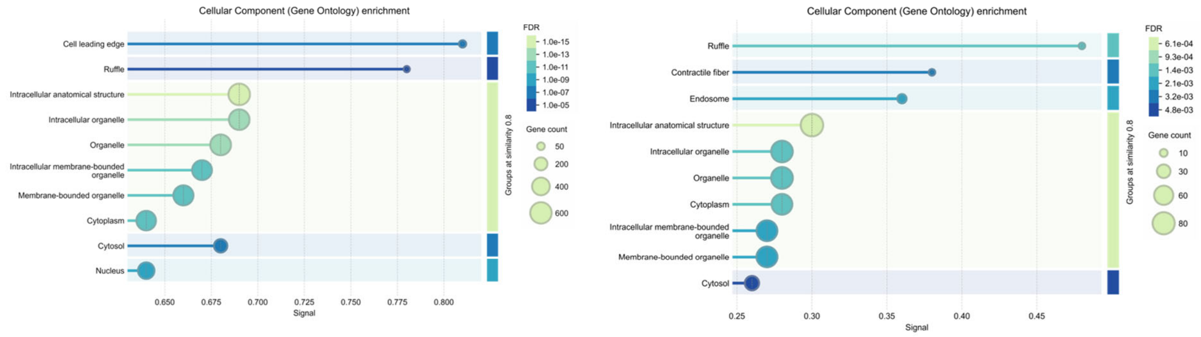

mRNA: unselected 6 mo upregulated

ceNET: 6mo UP

Figure S10. Cellular component (GO) enrichment results: 6-month upregulated mRNAs vs 6mo UP ceNET.

9-month upregulated mRNAs vs **9mo UP ceNET**

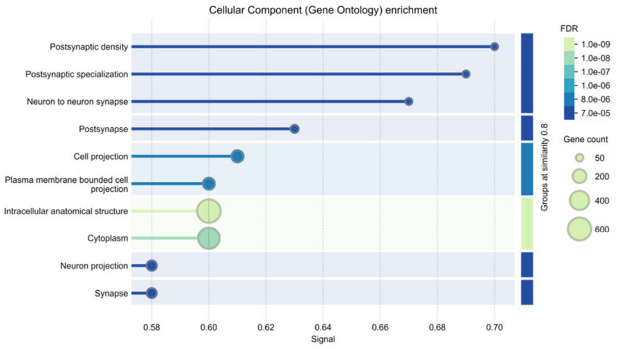

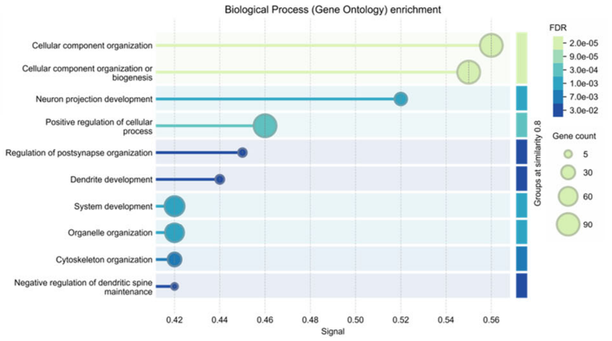

mRNA: unselected 9 mo upregulated

ceNET: 9mo UP

Figure S11. Cellular component (GO) enrichment results: 9-month upregulated mRNAs vs 9mo UP ceNET.

6-month upregulated mRNAs vs **6mo UP ceNET**

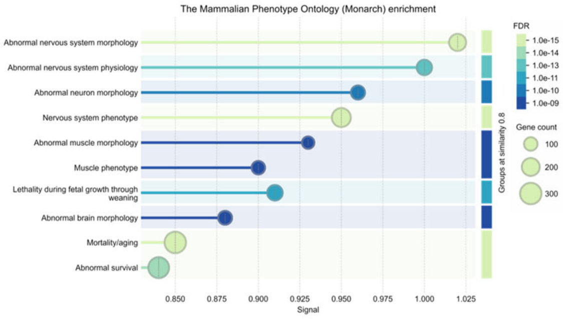

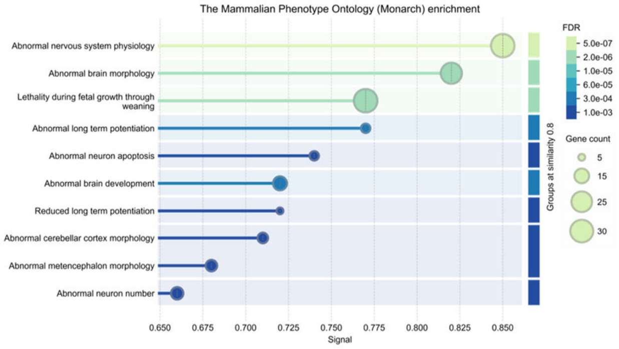

mRNA: unselected 6 mo upregulated

ceNET: 6mo UP

Figure S12. The mammalian phenotype ontology (Monarch) enrichment results: 6-month upregulated mRNAs vs 6mo UP ceNET.

9-month upregulated mRNAs vs **9mo UP ceNET**

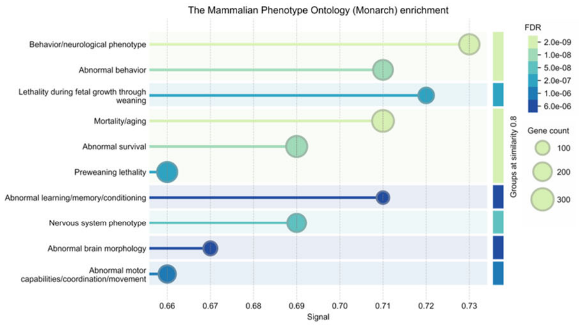

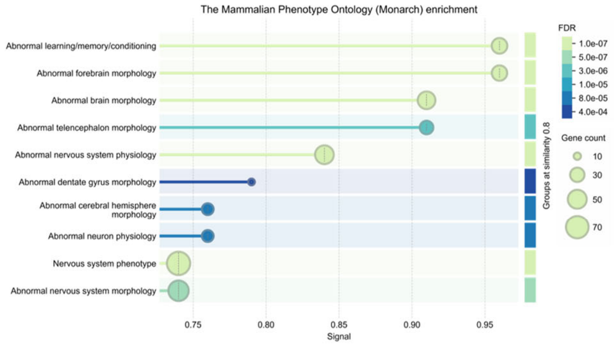

mRNA: unselected 9 mo upregulated

ceNET: 9mo UP

Figure S13. The mammalian phenotype ontology (Monarch) enrichment results: 9-month upregulated mRNAs vs 9mo UP ceNET.
